# Boron-Filled Hybrid Carbon Nanotubes

**DOI:** 10.1038/srep30495

**Published:** 2016-07-27

**Authors:** Rajen B. Patel, Tsengming Chou, Alokik Kanwal, David J. Apigo, Joseph Lefebvre, Frank Owens, Zafar Iqbal

**Affiliations:** 1 Materials Science and Engineering Program, New Jersey Institute of Technology, Newark, New Jersey 07102, USA; 2 Laboratory for Multiscale Imaging, Stevens Institute of Technology, Hoboken, New Jersey 07030, USA; 3 Department of Physics, New Jersey Institute of Technology, Newark, New Jersey 07102, USA; 4 Hysitron Incorporated, Eden Prairie, Minnesota 55344, USA; 5 Department of Physics, Hunter College, City University of New York, New York City, New York 10021, USA; 6 Department of Chemistry and Environmental Science, New Jersey Institute of Technology, Newark, New Jersey 07102, USA

## Abstract

A unique nanoheterostructure, a boron-filled hybrid carbon nanotube (BHCNT), has been synthesized using a one-step chemical vapor deposition process. The BHCNTs can be considered to be a novel form of boron carbide consisting of boron doped, distorted multiwalled carbon nanotubes (MWCNTs) encapsulating boron nanowires. These MWCNTs were found to be insulating in spite of their graphitic layered outer structures. While conventional MWCNTs have great axial strength, they have weak radial compressive strength, and do not bond well to one another or to other materials. In contrast, BHCNTs are shown to be up to 31% stiffer and 233% stronger than conventional MWCNTs in radial compression and have excellent mechanical properties at elevated temperatures. The corrugated surface of BHCNTs enables them to bond easily to themselves and other materials, in contrast to carbon nanotubes (CNTs). BHCNTs can, therefore, be used to make nanocomposites, nanopaper sheets, and bundles that are stronger than those made with CNTs.

Carbon is able to form a large number of unique nanostructures, such as closed hollow carbon cages otherwise known as fullerenes^1^,^2^,^3^,^4^,^5^. Among them, carbon nanotubes (CNTs), in particular, have attracted great interest, in part because of their exceptional mechanical properties. CNTs possess a Young’s modulus on the order of 270–950 GPa and a tensile strength of 11–63 GPa^6^,^7^,^8^,^9^,^10^,^11^,^12^, making them one of the strongest materials known. This strength, combined with the ductility of CNTs, makes them incredibly tough^13^,^14^,^15^. Unfortunately, because of their structural anisotropy and hollow cores, the radial mechanical properties of CNTs are quite poor and they do not perform as well under compression as they do in tension^16^,^17^,^18^,^19^,^20^.

Since the growth of extremely long CNTs is difficult^21^, CNTs are usually mixed with other materials to form composites^22^,^23^,^24^,^25^,^26^,^27^, or agglomerated into bundles or nanopaper sheets^8^,^28^, so that their mechanical properties can be exploited for macroscale applications. For composites, inadequate adhesion of the CNTs to the matrix material can result in poor mechanical properties, and in the case of bundles and nanopaper, weak bonding of adjacent nanotubes limits mechanical strength. Similarly, multiwalled CNTs (MWCNTs) with few available defect bonding sites have little wall to wall adhesion (causing superlubricity, which is actually useful for some applications)^29^, leading to the outer wall bearing the vast majority of any mechanical loading^6^. To mitigate these issues, the general approach has been to add functional groups or use radiation to induce defects after the production of the CNTs to bond adjacent walls of the nanotubes together^30^. While improving adhesion, functionalizing these carbon nanostructures can be sometimes detrimental to their intrinsic mechanical properties.

In this work, the weaknesses of CNTs mentioned previously are mitigated by growing MWCNTs around boron nanowires, with both materials grown in one step, to create boron filled hybrid carbon nanotubes (BHCNTs)^31^. Filled nanotubes themselves are not a new concept^32^,^33^,^34^, and neither is the filling of a nanotube with a boron based ceramic^35^. However, growing a nanotube around boron and demonstrating a radical alteration to the nanotube’s mechanical and electrical properties is novel. Here, boron nanowires fill the hollow core and increase the compressive radial strength of the MWCNTs. Furthermore, MWCNTs grown around boron nanowires possess a ‘corrugated’ structure, which may allow loading to transfer more effectively from the outer wall of a MWCNT. This uneven shape seems morphologically analogous to a 1-D form of crumpled graphene^36^. This structure may also allow for ‘interlocking’ to occur between adjacent nanotubes, making them far stronger when used in bundles or as nanopaper. The outer portion of the BHCNTs is shown to be similar to a highly defective MWCNT with a number of active binding sites, further facilitating the potential for applications as a high strength material. Interestingly, the filling process renders the BHCNT to be electrically insulating. An attempt has been made here to understand the electrical properties of BHCNTs using density functional calculations. The emergence of these unexpected properties differentiate BHCNTs from traditional filled carbon nanotubes, thus making them a *hybrid* form of nanotubes.

## Results and Discussion

**Figure 1:**
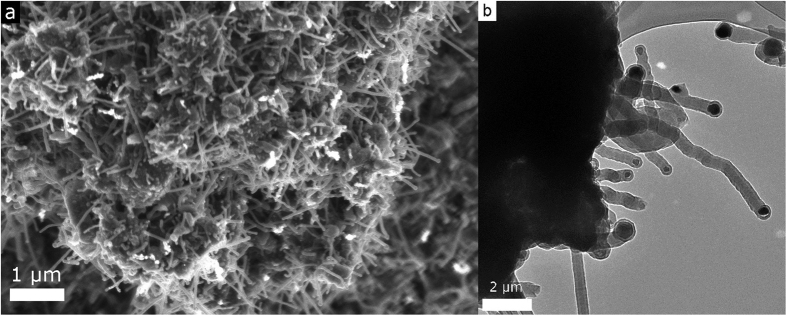
(**a**) Scanning electron microscopy (SEM) image of numerous boron filled hybrid carbon nanotubes (BHCNTs) protruding from the surface of a particle. (**b**) Transmission electron microscopy (TEM) image of BHCNTs, clearly showing they are not straight and have a bulbous tip from the catalyst.

Scanning Electron Microscopy (SEM) imaging (Fig. 1a) was conducted revealing that the material produced from the chemical vapor deposition reaction consisted mostly of 30–50 nm diameter nanowires that were several micrometers in length. The qualitative yield of the nanowires appeared to be excellent based on the SEM images, which showed that they were ubiquitous over the samples produced. Transmission Electron Microscopy (TEM) images (Fig. 1b) showed that the nanowires were corrugated, had numerous bends, and grew out of large particles. The presence of catalyst at the head of the nanowires reveals their growth is probably best described by a vapor-liquid-solid (VLS) or a vapor-solid-solid model (VSS)^37^,^38^.

**Figure 2:**
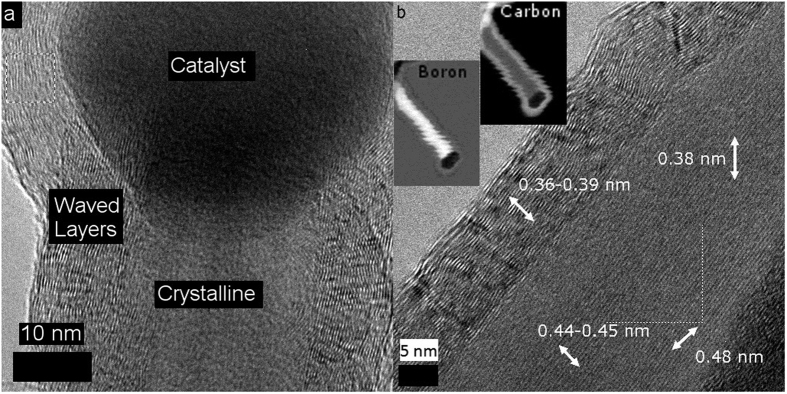
(**a**) High resolution TEM image of a catalyst particle within a BHCNT. Note the difference between the exterior, which encapsulates the catalyst, and interior of the structure. (**b**) High resolution TEM image of a BHCNT with labeled crystallographic spacings. The white arrows [not to scale] indicate the direction of the spacings. Insets: Boron and carbon electron energy loss spectroscopy maps from the BHCNT. Note the boron and carbon are mostly found on the inside and outside of the BHCNT, respectively.

High-resolution TEM images, shown in Fig. 2, revealed that the nanowire was actually two materials joined intimately together forming a core-shell, radial heterostructure. The outer part of each nanowire, including the area curled around the catalyst, is a layered, corrugated structure. Image analysis shows the interlayer spacing ranges from 0.36 nm to 0.39 nm, as confirmed using fast Fourier transform (FFT) analysis. Determining the exact spacing is difficult, most likely because the spacing varies significantly due to the uneven nature of the layers. The inner part of the nanoheterostructures appeared to be similar to the nanowires obtained from a closely related process using MgB_2_ without any dopant gases^39^. Using FFT analysis, the lattice spacings of the internal nanowires were found to be 0.48 nm, 0.38 nm, and 0.43 nm, and the crystal planes were offset at angles of 46.9°, 77.7°, and 54.6° from each other. More TEM and SEM images are provided in Supplementary Figure S1 with FFT analysis in Supplementary Figure S2.

Further analysis was performed using Electron Energy Loss Spectroscopy (EELS) in the scanning TEM mode, revealing the elemental composition and distribution in the nanowire (Fig. 2b, insets). The inside and outside of the nanoheterostructure was found to primarily consist of boron and carbon, respectively. There was some boron found in the exterior nanotube, roughly in a 1:9 ratio with the carbon, as evident from Supplementary Figure S3. This, combined with the information from bright field TEM imaging, shows that the nanowire heterostructure made in this process is a boron nanowire encased by a MWCNT, forming a BHCNT.

Raman spectroscopy was conducted to examine the structure of the carbon on the outside of a BHCNT. The Raman spectrum (Supplementary Figure S4) is consistent with that of a defected, somewhat disordered carbon structure on the outside of the nanowire. The D band was much higher in intensity in comparison to the G band when using both 632 and 532 nm wavelength laser excitation. In addition, there was a small downshift in the D band frequency when using a 532 nm laser, with a decrease in the intensity of the D band^40^,^41^,^42^,^43^. The Raman spectra are therefore consistent with that of a BHCNT consisting of a defective, disordered MWCNT encapsulating a boron nanowire. The TEM analysis roughly corroborates the Raman results, as it shows a high level of distortion in the interlayer spacing of the carbon, which is 0.36–0.39 nm, significantly larger than the typical spacing of 0.34 nm between carbon layers in a free-standing MWCNT^41^.

**Figure 3:**
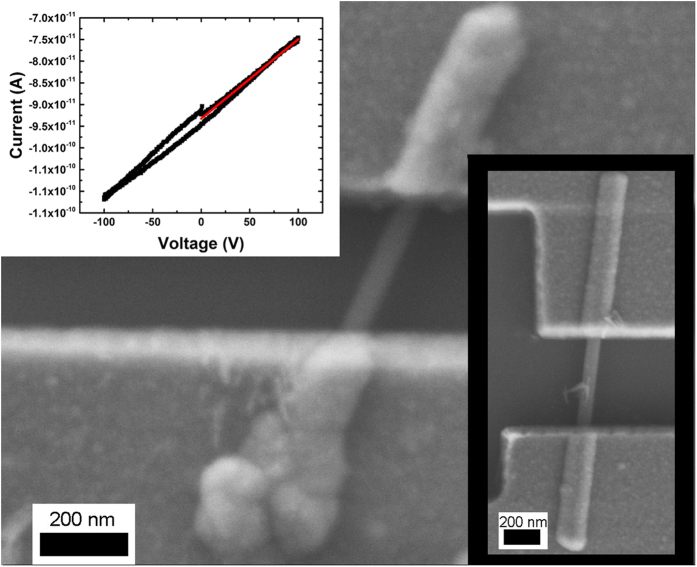
SEM image of a BHCNT undergoing a two point probe measurement to determine its electrical properties. (Top Left Inset) Typical current voltage curve measured from BHCNTs in the three experiments. The red line is a linear fit from 0 to 100 V. (Bottom Right Inset) Fabricated nanocontacts on a pure boron nanowire.

Since the carbon on the outside of the nanoheterostructure is different from that of a conventional nanotube, there was interest in comparing its electrical properties to that of MWCNTs. Also, to determine whether a BHCNT is useful as an electronic device because of its heterostructure nature, the interior boron nanowire was studied. Both the interior boron nanowire and the exterior MWCNT-like carbon were determined to be highly insulating using the Zyvex system (see Fig. 3 and Supplementary Figure S5). Conduction at the highest voltages occurred only because the underlying layer of silicon dioxide broke down and the current travelled through the silicon. For the interior boron nanowire, this result is not highly interesting, but for the exterior nanotube it is quite surprising. The insulating nature of BHCNTs was confirmed with the HP4140 B picoameter and the PI-85 PicoIndenter.

**Figure 4:**
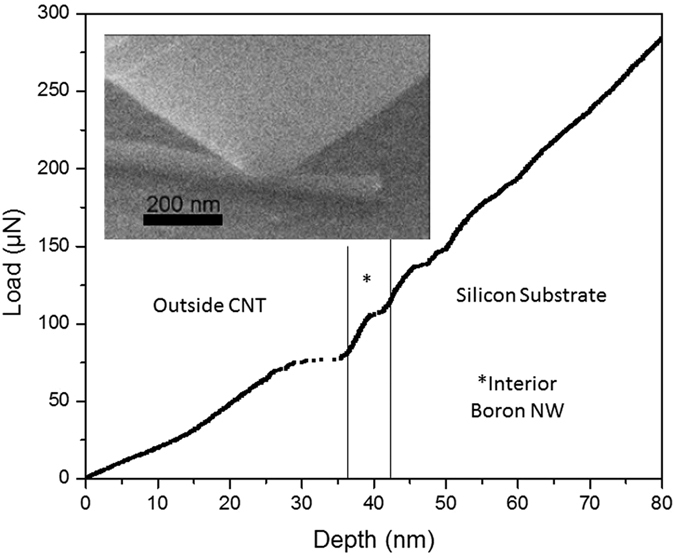
Load vs. displacement measurement of a BHCNT. (Inset) Image of live test of compressive mechanical properties of BHCNT using PI 85 PicoIndenter.

The PI 85 PicoIndenter was also used to determine the compressive radial mechanical properties of the BHCNTs, see Fig. 4 for a load-displacement curve from a radial compression test performed on a BHCNT. The first part of the curve demonstrates the stiffness and strength of the outer layer of BHCNT, which is greater than that of control CNTs (see Supplementary Figure S6). Furthermore, after the outer layer of the BHCNT fails, the interior boron nanowire is still able to support even greater amounts of loading with more rigidity. If one cuts off the outer layer of the BHCNT, mechanical properties of the interior wire can be probed directly, see Supplementary Figure S7. The supplemental section also has a video of a BHCNT as it stressed at 400 °C, which shows how much the heterostructure can bend under loading. Importantly, these measurements demonstrate that despite the large number of defects in the structure of the BHCNT, it still possesses advantageous mechanical properties and a great deal of flexiblity for a heterogenous material.

Compressive load vs. displacement measurements were performed on several BHCNTs, CNTs, and COOH functionalized CNTs, and the key results are summarized in Table 1 and Supplementary Figure S8. The testing primarily focused on three parameters, stiffness, failure loading at room temperature, and failure temperature under 30 μN of force (stiffness was determined by average deflection at 30 μN of force). The compression mechanics of single nanotube structures in the radial direction is quite complex, and remains a significant subset of the nanomechanics field of study. For this study, a simplified approach to the analysis of the raw mechanical data was used. This was accomplished by evaluating the displacement allowed by the various nanostructures under identical loading conditions. In all three parameters, the BHCNTs outperformed the CNTs and the COOH functionalized CNTs. (Since the BHCNTs were found to be defective using Raman spectroscopy, it was assumed they could become functionalized naturally once exposed to air or organic solvents, therefore, comparisons to both pristine and functionalized CNTS are useful. Furthermore, functionalized CNTs are often used in mechanical applications, making them all the more important for comparison.) While the COOH-CNTs were found to be overall superior to the unmodified CNTs, the BHCNTs outperformed both by a significant margin. The BHCNTs were on average 30% stiffer than the pristine CNTs, and their outer carbon shells could support 2.5 times more force before failing. The inner nanowire was able to further resist deformation by another 25 μN, making the entire structure over 3 times stronger than a pristine CNT. The superiority of the BHCNTs continued into elevated temperatures. Many of the pristine CNTs failed at room temperature with 30 μN force, and all of the ones tested at 100 °C failed. The functionalized CNTs and the BHCNT outer shell survived 30 μN of force until 200 °C. The interior boron nanowire of the BHCNT, however, was resistant to failure until the highest accessible temperature in the study, 400 °C.

The experimental results indicate the multi-layered carbon nanotubes that encapsulate the boron nanowires could have many vacancies and are boron doped. Furthermore, the structure of the carbon tubes is quite disordered. To elucidate how boron doping and vacancies effect the electronic and geometric structure of the MWCNT, modeling of double-walled carbon nanotubes (DWCNT) was performed. Admittedly, a two layered DWCNT does not correspond to the many layered MWCNT observed here. However, modeling a MWCNT of many layers is well beyond the limits of available computer time on a supercomputer. In fact, a literature search revealed only one report of tight binding modeling of a two layered armchair MWCNT^44^ and no reports of density functional calculations of any MWCNTs. It is possible that models of a DWCNT doped with boron and/or having vacancies could account for some of properties observed in a many layered MWCNT. Zig-zag DWCNTs without defects, with vacancies, and with boron doping were initially optimized using the semi-empirical PM3 method. The output of these calculations were then used as the input to a density functional calculation at the B3LYP/6-31G* level so that the minimum energy structure could be determined, see Supplementary Figure S9.

Figure S9a shows a side and end view of the optimized structure of an undoped DWCNT having no vacancies. The outer tube and inner tube are zig-zag in structure with chiralities of (14,0) and (10,0), respectively. The model shows that defect free DWCNTs have an ordered arrangement of carbons. Figure S9b depicts the optimized structure of the same DWCNT doped with 10 boron atoms in the outer layer. The calculations indicate that there is considerable disorder and distortion of the structure of the boron doped carbon DWCNT. Thus, they suggest that the presence of boron atoms in the outer layer is partially responsible for the observed disruption of the structure. However, the calculations indicate that the presence of boron in the outer layers is not responsible for the observed insulating properties of the BHCNTs. For the undoped and doped DWCNT, the band gap at the center of the Brillioun zone was calculated to be 0.62 eV and 0.52 eV, respectively. The effect of vacancies in the outer layer was also investigated. Figure S9c displays the optimized structure of the DWCNT having 10 vacancies, which also has considerable corrugation. This suggests that the presence of the vacancies could also contribute to the disruption of the structure. Results similar to this have been reported for graphene, which also shows crumpling with the addition of defects^45^. The calculated band gap of the DWCNT having vacancies was 0.59 eV, only slightly less than the DWCNT without vacancies. This model of the two layer DWCNTs indicates that neither boron doping nor the vacancies in the outer layer contribute to the observed insulating behavior of the BHCNT, since the calculated band gaps of the 3 DWCNTs indicate that they are all semiconducting. The significance of the difference in the magnitude of the band gaps is unclear. The relative stability of the different structures is assessed by calculating the binding energy (BE) given by,


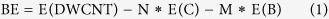


where E(DWCNT) is the total calculated energy at the minimum energy structure of the DWCNT, N is the number of carbon atoms in the structure, and M the number of boron atoms, E(C) and E(B) are the total electronic energy of a single carbon and boron atom, respectively. The results show that the unaltered DWCNT is the most stable of the structures having a BE of 628.6020 AU. The least stable is the DWCNT with 10 vacancies having a BE of 564.6797 AU. The calculated BE of the 10 boron doped DWCNT is 611.7802 AU.

## Conclusions

A new type of nanoheterostructure, a boron carbide hybrid nanowire (BHCNT), has been synthesized with a one step process. A BHCNT can be described succinctly as a boron nanowire surrounded by a corrugated MWCNT doped with 10% boron. (While highly distorted and possessing some novel properties, as the material on the outside of the BHCNT is still a layered carbon, it is best described as a special case of a MWCNT.) Due to the highly defective nature of the material, it is possible other elements may be present in the BHCNT in small quantities. This is a rare instance where the nanoheterostructure was grown using constant growth conditions throughout an experimental run^46^. This could have implications for the future synthesis of other nanoheterostructures, as they are usually made by altering the growth conditions of a material *in situ*^47^. BHCNTs are a novel form of boron carbide and should be differentiated from homogenous boron carbide nanowires^48^,^49^. Moreover, the outer MWCNT in the structure is surprisingly electrically insulating, suggesting that the BHCNTs can also be considered to be a new carbon form. Although 10% doping of the MWCNT layer with boron is observed in the EELS data, it is unlikely to significantly lower the electrical conductivity. The origin of the insulating properties of the BHCNT is difficult to determine and therefore remains unresolved. While it is known the addition of defects via functionalization, doping, and vacancies reduces the electrical conductivity of CNTs, the dramatically reduced conductivity observed in this work is quite surprising and exceeds that of known materials composed mostly of carbon.

The radial mechanical properties of the BHCNT were found to be superior to those of MWCNTs. Furthermore, by using boron as a filler, the molecular weight is kept low, suggesting the possibility of using BHCNTs with enriched boron for radiation shielding applications. Radial compressive stress strain tests are a useful starting point, as nanotubes are particularly weak under this type of loading. Future investigations will examine other scenarios, such as three point flexure tests and axial tension/compression. Even if individual BHCNTs are found to be weaker than or comparable to MWCNTs under those types of stresses, the overall advantages they possess may make them suitable for many applications. Comprehensive modeling will also be a useful tool for in depth analysis of the mechanical properties of BHCNTs, especially to examine different failure conditions^50^. Future experiments will also focus on creating nanopaper, bundles, and composites using BHCNTs and comparing them to those made using conventional CNTs, where maximization of the mechanical properties will be the prime objective. The corrugated outer layer, in conjunction with the large number of bonding sites, may allow the nanotubes to bind better to matrix materials, increasing the strength of composites. These same properties may also allow BHCNTs to bind to each other more easily, allowing them to form stronger bundles and nanopaper.

## Methods

### Synthesis

The method of synthesizing BHCNTs is a modification of a process used to make a number of different boron nanostructures^51^,^52^,^53^. First, 50 wt% MgB_2_ (Alfa Aesar), 30 wt% nanoNiB (prepared in a manner following references)^53^,^54^ and 20 wt% of mesostructured hexagonal framework MCM–41 zeolite powder (Sigma Aldrich) were mixed and reduced in particle size by grinding using a mortar and pestle. Typically, 0.02–0.1 grams of the mixture were added and ground in an agate mortar for about an hour to ensure that the powder was well mixed. It was then ground further for several hours in a rotary mixer using cylindrical ceramic pieces as milling media. Finally, the mixture was loaded in a ceramic boat that was placed in the quartz reactor of the chemical vapor deposition (CVD) system (Supplementary Figure S10). The quartz tube was pumped down to 10^−3^ torr and heated to 950 °C at a rate of 10 °C/min under argon and methane flows of 100 sccm (standard cubic centimeters per minute) and 10 sccm, respectively. The temperature was held at 950 °C for 60 minutes before the furnace was switched off and the reaction tube was allowed to cool down to room temperature under flowing argon.

### Electron Microscopy

Scanning Electron Microscopy (SEM): SEM images were obtained with a VP-1530 Carl Zeiss LEO (Peabody, MA) field emission scanning electron microscope. The samples were mounted on aluminum stubs using double-sided carbon tape.

Transmission Electron Microscopy (TEM): A FEI CM-20 field emission gun S/TEM equipped with a Gatan Enfina parallel electron energy loss spectrometer was used. Samples were suspended in ultra-pure methanol at 1 wt% concentration, and a 1 μL drop of the solution was placed on a lacey carbon TEM grid placed on a filter paper. The TEM grid was then put into a vacuum oven to dry at 80 °C. Electron energy loss spectroscopy Spectrum Imaging (EELS SI) data was collected in conjunction with Scanning TEM (STEM) dark field imaging to fully characterize the nanomaterial. To overcome sample-drifting, an active drift correction was used while collecting the data. To effectively collect EELS data, a 6.7 mrad convergence and 13 mrad collection angle were used. EELS data was analyzed using EELS analysis plugins in Gatan DigitalMicrograph software.

### Raman Spectroscopy

Raman spectra were obtained using a confocal Horiba-Jobin Yvon LabRam microRaman spectrometer with a 20 mW laser source emitting at a wavelength of either 632 nm or 532 nm, focused to a spot size of 10 μm with a 10x lens. The spectra were recorded with a 200 μm slit and 2 × 15 sec acquisition time.

### Electrical Property Measurements

Electrical measurements were made using the aforementioned SEM microscope with a Zyvex Nanomanipulator S100, which could perform current voltage (IV) sweeps on individual nanowires. BHCNTs were deposited on a silicon substrate with a 100 nm thermally grown oxide layer to electrically isolate the test specimens. To ensure contact, leads were deposited on individual nanostructures. Furthermore, to explore any possible devices which come from the heterostructure, the electrical properties of the interior boron nanowire were also studied. This was straightforward because the interior boron nanowires without a carbon layer could be synthesized by performing the described CVD reaction without methane gas. To confirm the results from the Zyvex system, electrical measurements with pads connected to leads deposited on the BHCNTs and pure boron nanowires were conducted with a probe station in a dark box using an HP4140B picoameter. These results were further corroborated with a PI 85 SEM PicoIndenter which directly contacted the BHCNTs.

### Mechanical Property Measurements

*In situ* radial mechanical property measurements of individual BHCNTs were made using a PI 85 SEM PicoIndenter equipped with sample heating capabilities for elevated temperature measurements. The PI 85 was installed on a Versa 3D FIB/SEM (FEI Company) to enable identification of suitable nanotubes, alignment with the compression probe, and direct observation of the compression tests with the SEM imaging capabilities. Pristine and COOH doped MWCNTs were purchased from Cheaptubes as control samples. The control nanotubes had diameters and lengths of roughly 30–50 nm and 0.5–2 μm, respectively. Since dimensions of the nanomaterial are obviously a factor in its mechanical properties, only nanomaterials with ~50 nm diameter and ~2 μm length were probed. Temperature variable strength measurements were performed by observing if these materials would fail under 30 μN of force at the following temperatures: room temperature, 100 °C, 200 °C, 300 °C, and 400 °C. The primary source of experimental error in the mechanical property measurements would stem from the uncertainty surrounding the tip-sample and sample-substrate contact. Additionally, the sample is so thin that the supporting substrate may also be part of the measured response. This was mitigated by testing all samples under the same conditions and by testing many specimens from the same sample to ensure repeatability.

### Theoretical Modeling

To better understand the effect of boron doping and the presence of vacancies on the structural and electronic properties of BHCNTs, density functional (DFT) calculations were employed to calculate the minimum energy structure of a two layered multiwalled nanotube having 119 atoms. This structure was then compared to a MWCNT doped with boron or having vacancy defects to make it comparable to the MWCNT found on the outer layer of the BHCNT. The calculations of the optimized structure were carried out at the B3LYP/6-31G* level on the Excalibur supercomputer at the Army Research Laboratory.

## Additional Information

**How to cite this article**: Patel, R. B. *et al.* Boron-Filled Hybrid Carbon Nanotubes. *Sci. Rep.* **6**, 30495; doi: 10.1038/srep30495 (2016).

## Supplementary Material

Supplementary Information

## Figures and Tables

**Table 1:** Data summarizing load vs. strain measurements of BHCNTs and control CNTs.

Material	Fail Loading at Room Temp	Fail Temp at 30 μN	Stiffness in comparison to CNT
BHCNT-Outer Layer	75 μN	200 °C	130%
BHCNT-Inner Boron Wire	100 μN	400 °C	NA
CNT	30 μN	Room Temp	100%
CNT-COOH	50 μN (30 nm depth pen.)	200 °C	110%
