# Supplementary material for: Boron-Filled Hybrid Carbon Nanotubes
Source: Sci Rep. 2016 Jul 27;6:30495. doi: 10.1038/srep30495 (PMC4962039; doi:10.1038/srep30495)
Supplement: Supplementary Information [file srep30495-s1.pdf]

Boron-Filled Hybrid Carbon Nanotubes

1.) Rajen B. Patel*

Materials Science and Engineering Program, New Jersey Institute of Technology, Newark, New Jersey 07102

2.) Tsengming Chou

Laboratory for Multiscale Imaging, Stevens Institute of Technology, Hoboken, New Jersey 07030

3.) Alokik Kanwal, 6.) David Apigo

Department of Physics, New Jersey Institute of Technology, Newark, New Jersey 07102

4.) Joseph Lefebvre

Hysitron Incorporated, Eden Prairie, Minnesota 55344

5.) Frank Owens

Department of Physics, Hunter College, City University of New York, New York City, New York 10021

7.) Zafar Iqbal

Department of Chemistry and Environmental Science, New Jersey Institute of Technology, Newark, New Jersey 07102

Video Caption: This video shows a live compression test on a BHCNT using a PI 85 Picoindenter at 400 ^{o}C.

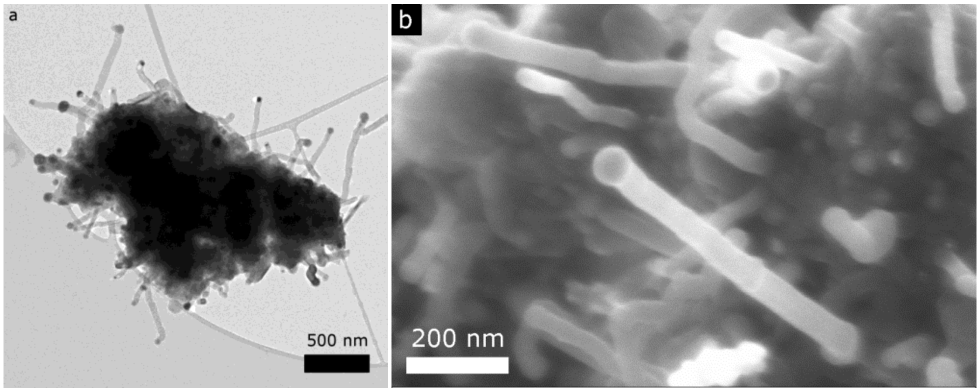

Figure S1 (a) TEM image of BHCNTs protruding from catalyst particle and (b) SEM image of same. Note the nanostructures have a bulbous tip and appear to be heavily corrugated.

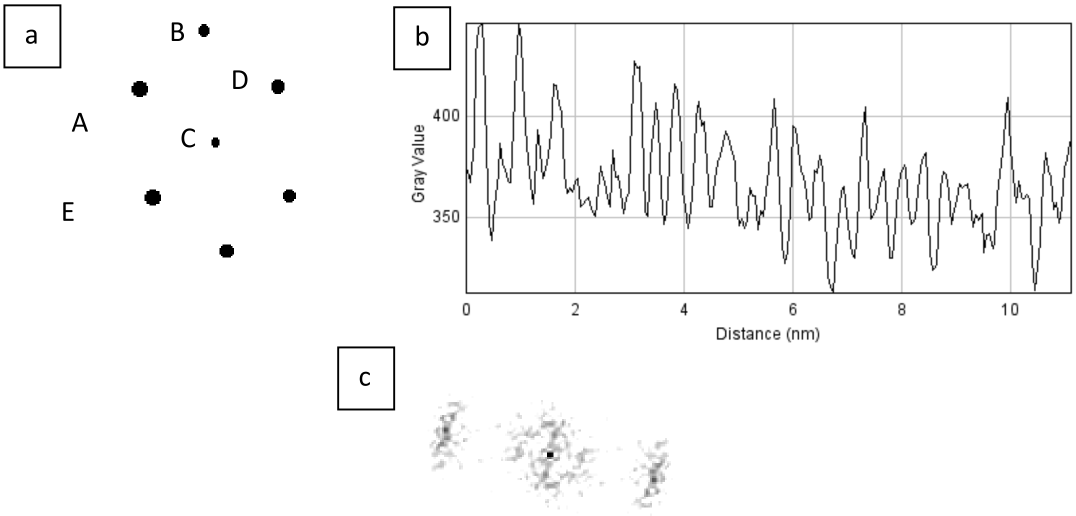

Figure S2 (a) Masked image of FFT pattern of interior nanowire with labeled points, making analysis easier. The following were measured using the ImageJ software suit, EC=DC=.48, AC=.44 to .45, BC=0.38, $\angle ACB=46.9^{o}$, $\angle BCD=54.6^{o}$, $\angle ACE=77.7^{o}$. (b) Intensity profile plot and (c) masked FFT analysis of the outer walls of the heterostructure, showing a spacing of approximately 0.36 nm to 0.39 nm.

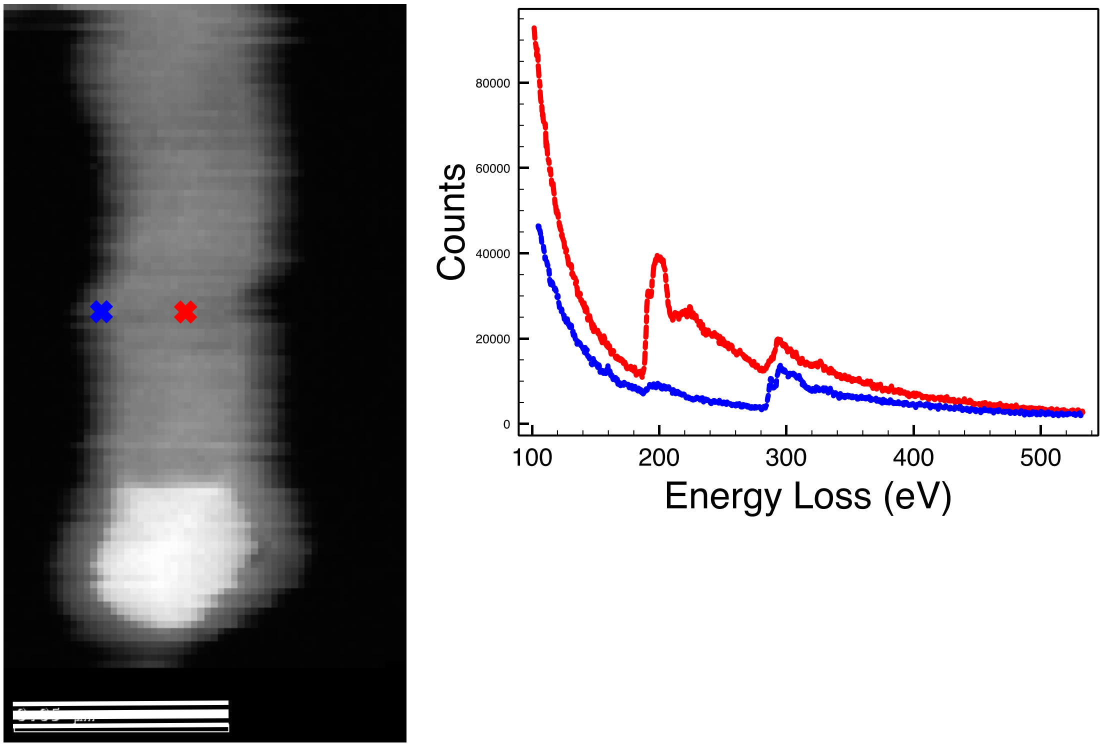

Atomic Ratio (per Carbon)	B	C
Center	1.55 ± 0.091	1.00 ± 0.000
Shell	0.11 ± 0.016	1.00 ± 0.000

Figure S3 EELS analysis of BHCNT. The image marker is 50 nm, the red line is the EELS measurement from the center part of the nanowire, and the blue line is from the shell. The energy loss edge at 188 eV and 284 eV indicates boron and carbon, respectively. The corresponding convergence angle is 6.7 mrad and the collection angle of the EELS spectrometer is 13 mrad. Data was processed and analyzed using EELS Quantification plugin in Gatan DigitalMicrograph software. The relative atomic ratio of carbon and boron was calculated and the calculation was normalized based on the number of carbon atoms.

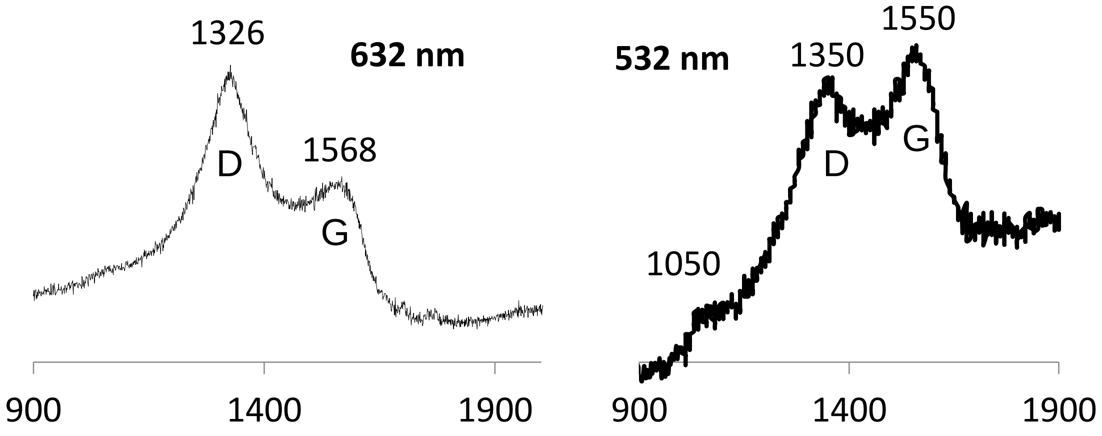

Figure S4 Raman spectroscopy of BHCNTs using 632 nm and 532 nm excitation lasers. The ratio of the intensity of the D and G bands is indicative of a highly defective structure.

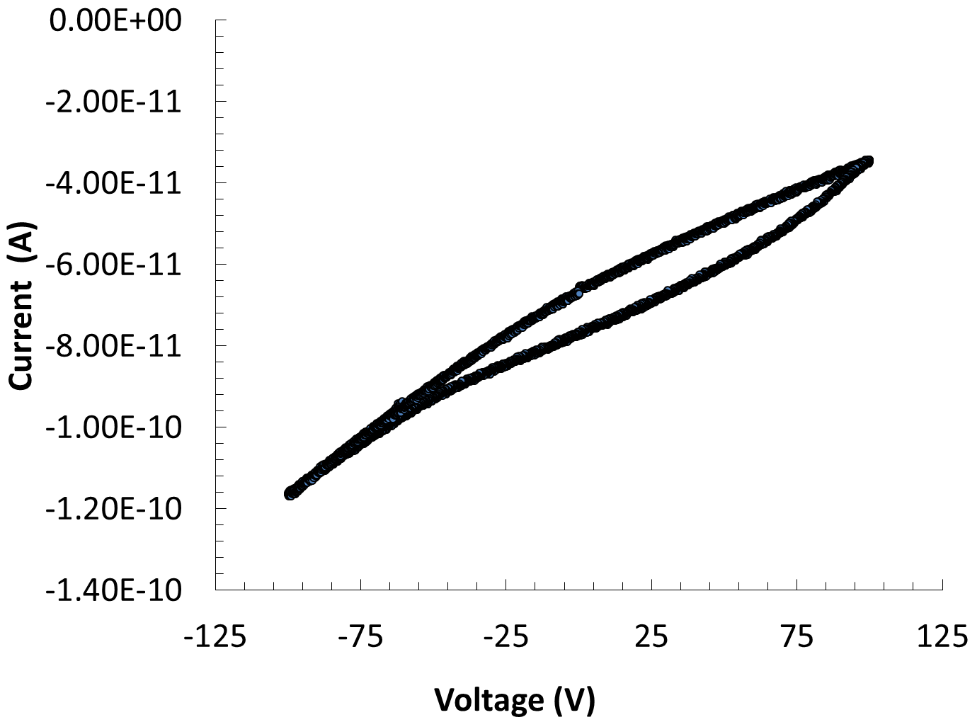

Figure S5 Typical IV curve measured from pure boron nanowires in the three experiments. Note that at this voltage there is a breakdown of the underlying silicon dioxide layer, which means that the measured current is not of the sample but of the substrate. This gives an indication of the extremely low conductivity of the boron nanowires

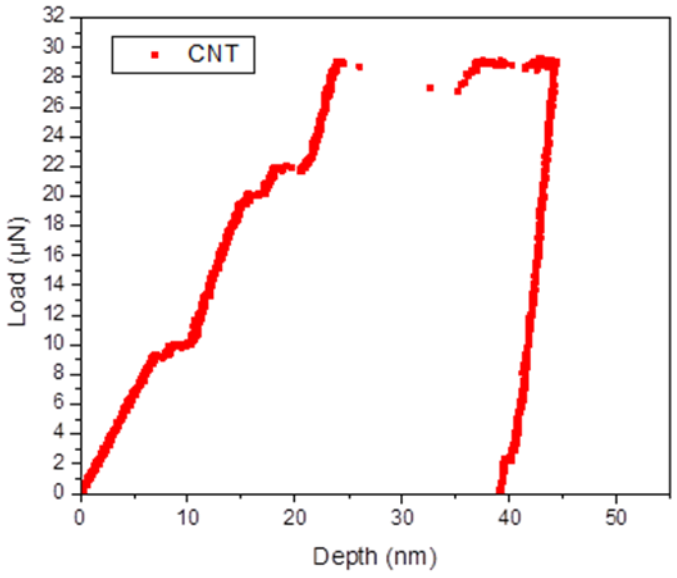

Figure S6 Load-displacement curve for a pristine MWCNT which acted as a control in this study. Note that failures appears to occur at a loading of roughly 30 μN at room temperature. This result was relatively repeatable.

a

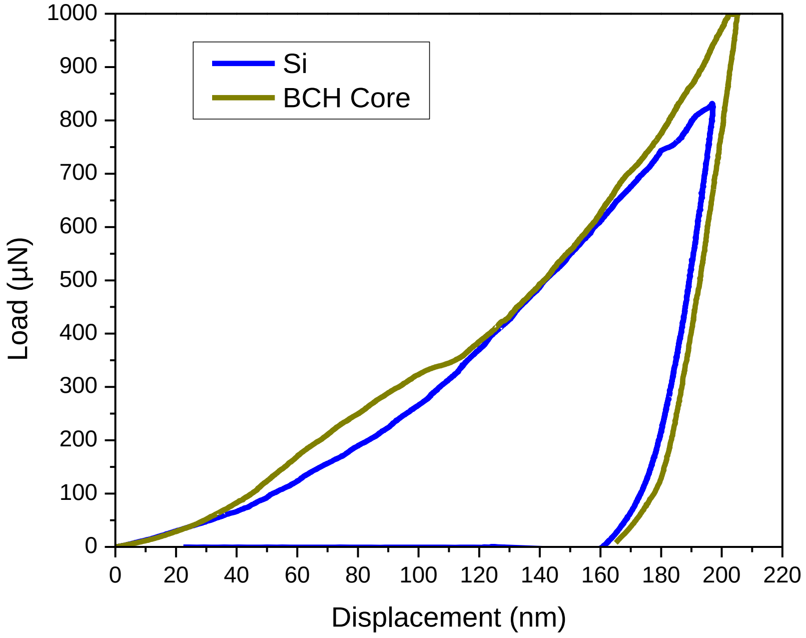

b

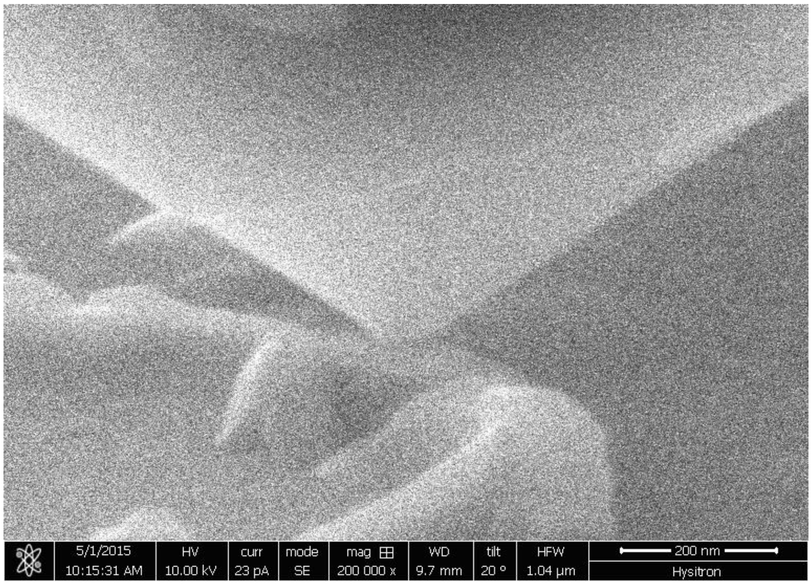

Figure S7 (a) Load-displacement curve for interior boron nanowire and (b) image of tested structure. All tests were down in compression in the radial direction.

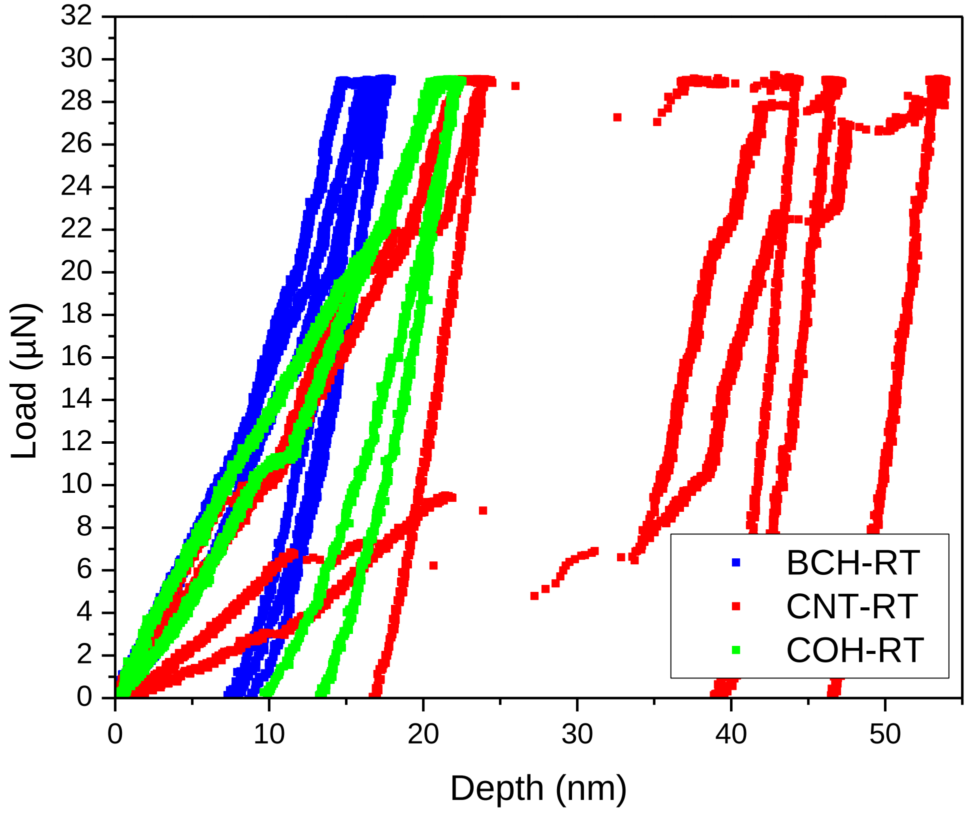

Figure S8 Summary of raw mechanical data for tests which did not cut through the nanostructures. Several experiments were conducted for each material, and the BHCNT (blue) was found to stiffer than the other materials. The BHCNT material also appeared to have less variation in its strength.

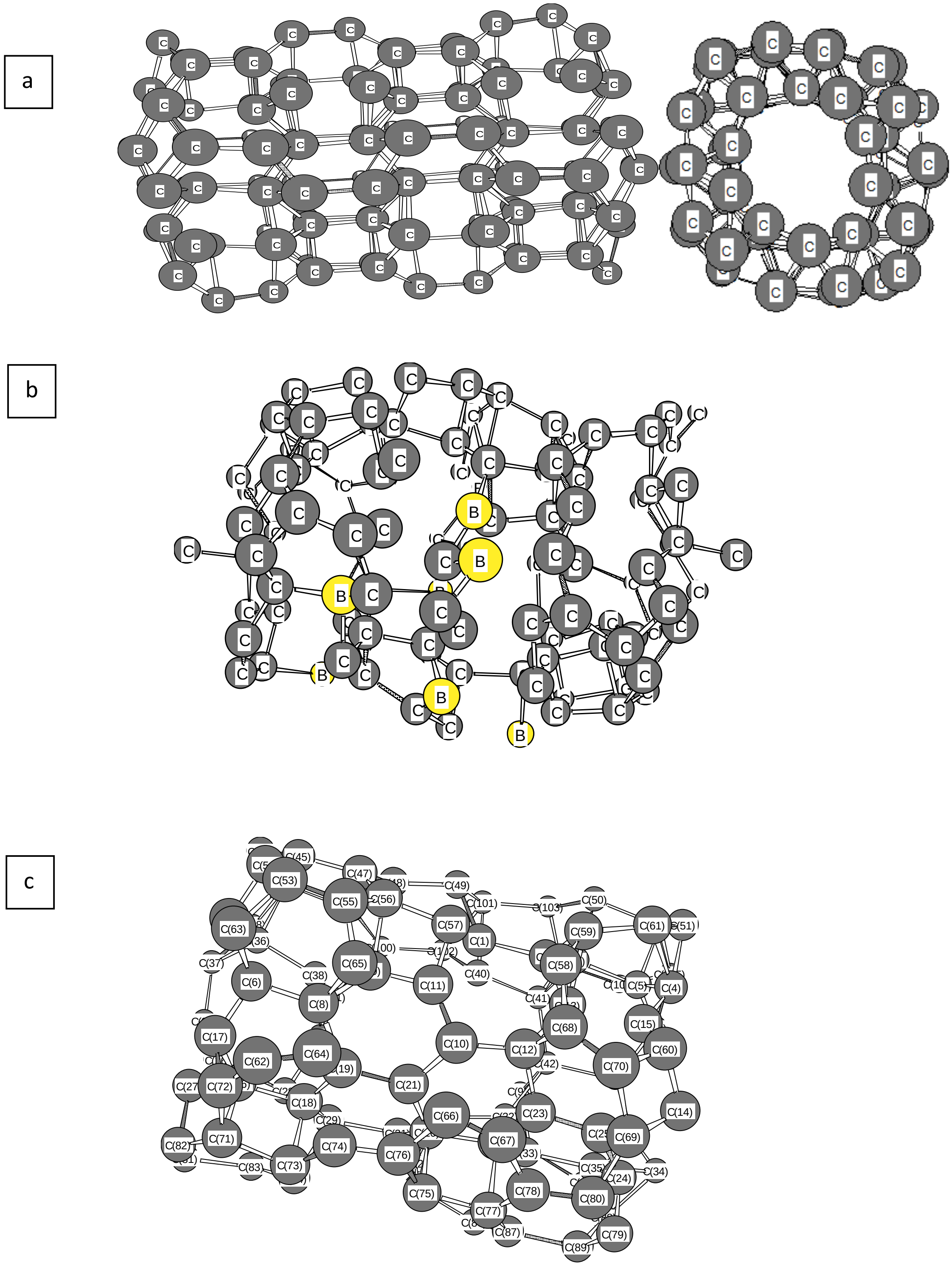

Figure S9 (a) Pristine MWCNT. (b) Boron as substitutional defects in the structure of a double walled MWCNT. (c) Vacancy defects in the structure of a MWCNT.

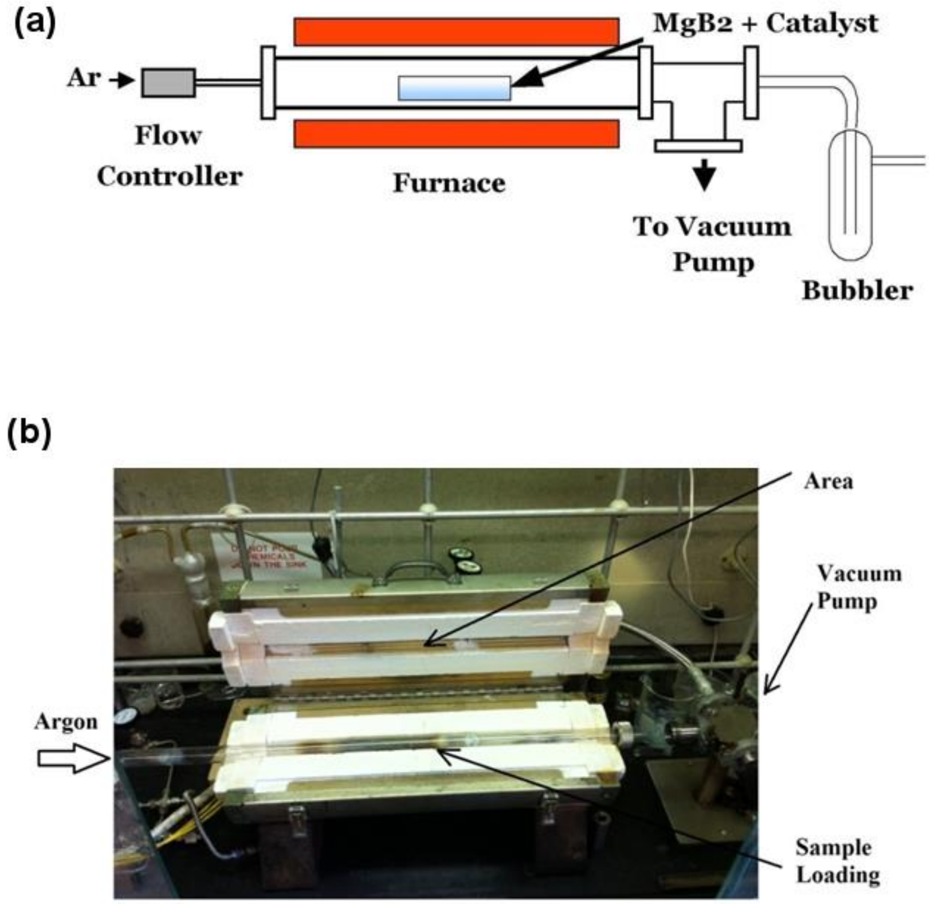

Figure S10 (a) Model and (b) picture of experimental set up used. This is a relatively standard chemical vapor deposition set up, which can be easily scaled and is quite flexible in the materials it can synthesize.
